# Human iPSC-Derived Neural Models for Studying Alzheimer’s Disease: from Neural Stem Cells to Cerebral Organoids

**DOI:** 10.1007/s12015-021-10254-3

**Published:** 2022-02-02

**Authors:** Martin Barak, Veronika Fedorova, Veronika Pospisilova, Jan Raska, Simona Vochyanova, Jiri Sedmik, Hana Hribkova, Hana Klimova, Tereza Vanova, Dasa Bohaciakova

**Affiliations:** 1 Department of Histology and Embryology, Faculty of Medicine, Masaryk University Brno, Brno, Czech Republic; 2 International Clinical Research Center, St. Anne’s Faculty Hospital Brno, Brno, Czech Republic

**Keywords:** iPSCs, Neural differentiation, Alzheimer’s disease, In vitro differentiation, Neural stem cells, Neural progenitors, Neurons, Astrocytes, Microglia, Cerebral organoids

## Abstract

During the past two decades, induced pluripotent stem cells (iPSCs) have been widely used to study mechanisms of human neural development, disease modeling, and drug discovery in vitro. Especially in the field of Alzheimer’s disease (AD), where this treatment is lacking, tremendous effort has been put into the investigation of molecular mechanisms behind this disease using induced pluripotent stem cell-based models. Numerous of these studies have found either novel regulatory mechanisms that could be exploited to develop relevant drugs for AD treatment or have already tested small molecules on in vitro cultures, directly demonstrating their effect on amelioration of AD-associated pathology. This review thus summarizes currently used differentiation strategies of induced pluripotent stem cells towards neuronal and glial cell types and cerebral organoids and their utilization in modeling AD and potential drug discovery.

**Graphical abstract:**

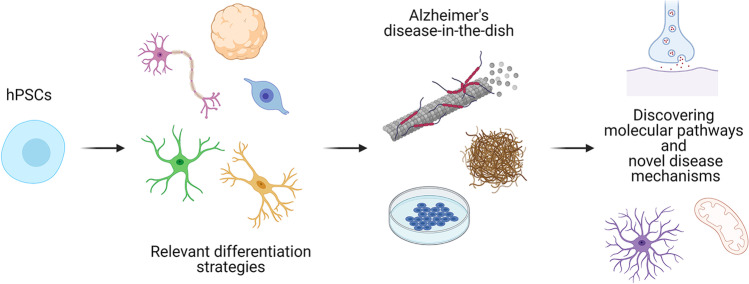

## Introduction

Since their discovery, pluripotent stem cells and their differentiated progeny served as models for studying mechanisms of human neural development in vitro. Since the advent of induced pluripotent stem cell (iPSC) technology and CRISPR/Cas9 gene editing, they have become an excellent and unique tool to not only model human CNS-related diseases in the dish but also served as a resource for the drug discovery effort. This is an especially relevant feature for the field of Alzheimer's disease (AD), where this treatment remains unavailable. Notably, over 60 studies have thus far been published that used iPSC-derived neural models to study AD in vitro. This review summarizes these studies and provides a unique view of AD-iPSC-based studies from the "differentiated cell type" perspective. Specifically, we provide a comprehensive summary of currently used approaches of differentiation of iPSCs to neural stem cells, neurons, glia, and organoids, including direct transdifferentiation approaches. At the same time, we summarize in vitro iPSC-based studies performed thus far on AD and highlight their key findings. Additionally, our review also contains a comprehensive table where all studies are listed based on the cell type, the type of mutation studied, and the main outcomes of the study. It could thus be used as a useful resource for researchers studying AD.

## Current Neurodifferentiation Strategies

The development of the central nervous system in vivo is governed by a tightly regulated balance between neural stem/progenitor/precursor cell (NPC) proliferation and differentiation towards mature cell types (reviewed in [1, 2]). During early embryonic development in vivo (for details on the concept of neural induction, see BOX 1), the neural tube forms via primary and secondary neurulation (reviewed in [3]). During primary neurulation, a process via which the neural tube is formed in the head and trunk regions of the body, ectoderm becomes sequentially specified to give rise to the epidermis, the neural plate (neuro-ectoderm), and the neural tube. During the secondary neurulation, which occurs specifically in the caudal region in all vertebrates, including humans, condensed mesoderm directly transitions to (neuro)epithelium and forms a neural tube. Irrespectively of the means of differentiation, the neural tube is, at this stage, composed of multipotent NPCs, which further in the development become more restricted. With respect to their potency to generate neurons, astrocytes, or oligodendrocytes, they are referred to as “neuronal “, “astrocyte “, and “oligodendrocyte “ precursors, respectively. Importantly, in vitro isolation and propagation of NPCs from the developing and adult rodent CNS has provided an essential tool to study the biology of NPCs and lineage differentiation potential (rat models reviewed in [4, 5], mouse models reviewed in [6]). Additionally, it also served as a basis for the induction of neural differentiation from pluripotent ES cells.

**Fig. 1:**
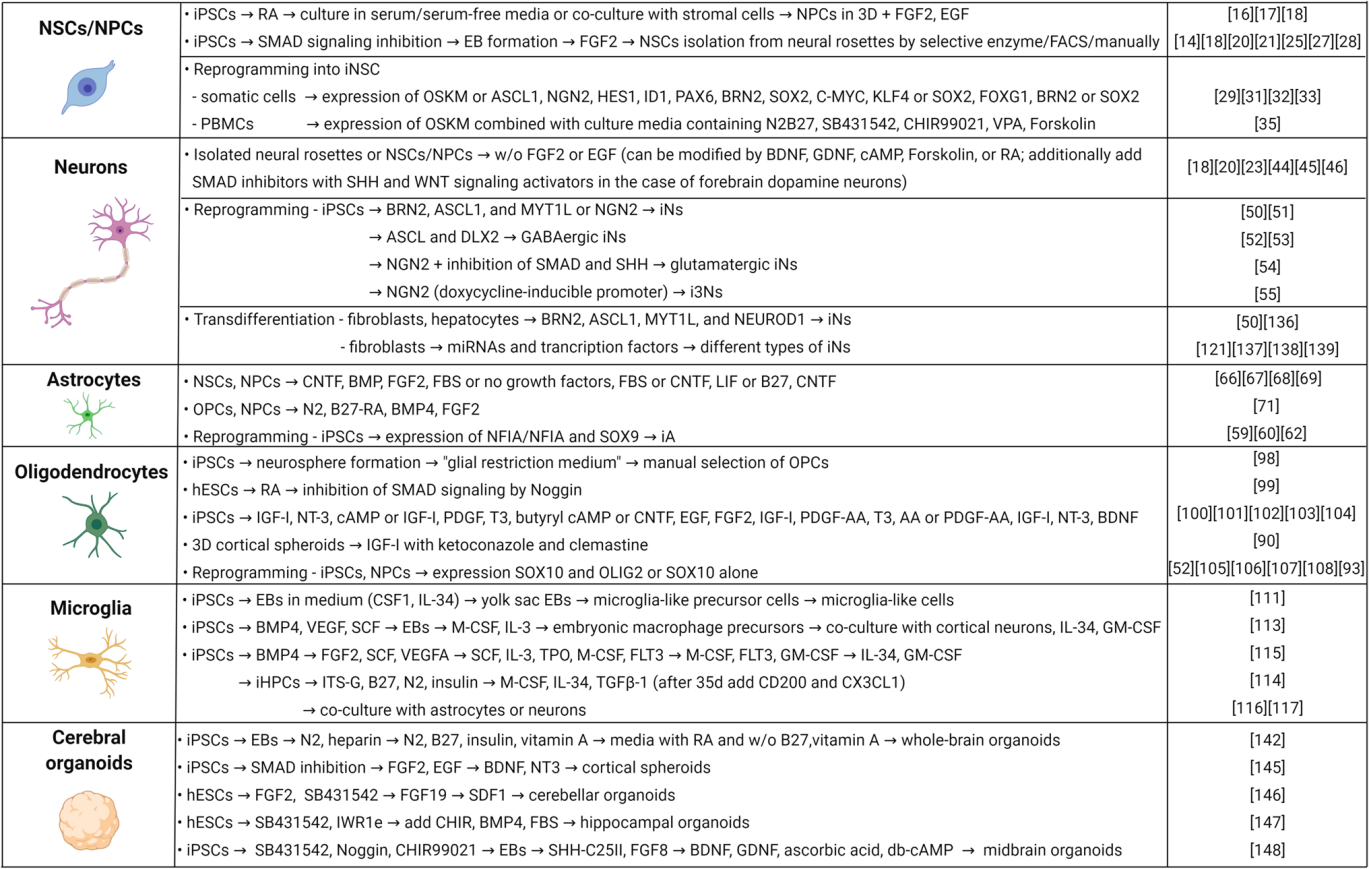
Differentiation protocols. Summary of neurodifferentiation strategies to generate specific cell types of the central nervous system from stem cells. For each strategy, we list major growth factors, small molecules, and other reagents that must be added to the cell culture media. Media also often contain N2 and B27 Supplements. Thus, for complete protocols, refer to the respective references listed in the last column

Currently, numerous differentiation protocols exist for the differentiation of neural cell types from human iPSCs, and these methods are comprehensively summarized in Fig. 1. They employ several strategies, which usually aim to mimic the in vivo developmental steps: the formation of the neuroepithelium, specialization of neural stem cells, which further differentiate towards neural progenitors and more mature cell types. The differentiation is usually achieved via the combination of specific cell culture media, growth factors, and small molecule inhibitors sequentially added to the culture media. Additionally, there are also differentiation protocols that are based on inducible overexpression of specific transcription factors that direct the differentiation towards a specific cell type. These are becoming increasingly common as they generate a relatively uniform population of the differentiated cell type of interest. Additionally, all in vitro strategies can also be divided into 2D and 3D methods, where 2D generate relatively simple and easy to characterize cell populations. On the other hand, 3D differentiation models are more functionally complex and more adequately mimic the developmental processes [7]. In the following chapters, we will summarize the necessary steps that are followed in each differentiation strategy. A special chapter is also dedicated to direct transdifferentiation strategies that might be especially important to consider when mimicking neurodegenerative diseases in vitro.

BOX 1: Concept of neural induction in vivo

**Table:** 

In the developing embryo, cell fate determination represents the ultimate decision to initialize the formation of a specific structure. The pivotal experiments studying the onset of the nervous system development were carried out by Spemann and Mangold in amphibian embryos nearly 100 years ago. In their work, they introduced a concept of induction, which shows that the developing ectoderm relies on mesodermal signals to induce neurulation [8]. The mediators of the induction were studied by Saxén and Toivonen, who defined two gradients of “neuralizing” and “mesodermalizing substances” in the inductor tissue that influence the formation of the neural tube [9]. Later on, molecules NOGGIN, FOLLISTATIN, and CHORDIN were identified to play a role during neurulation [10–12]. The follow-up studies indicated that the function of these molecules lies in the inhibition of the bone morphogenic protein (BMP) signaling pathway and the suppression of BMP was found to be the key primary step in the neural-fate acquisition of the early ectoderm (reviewed in [13])	

### Differentiation of Pluripotent Stem Cells Towards Neuroectoderm and Neural Stem Cells

Early methods to direct the differentiation of iPSCs to neural fates used less defined approaches than currently used protocols. Treatment of human embryonic stem cells with retinoic acid—RA [14], sequential culture in serum and serum-free media [15], or co-culture with specific stromal cell lines such as PA6 [16] were commonly used techniques to gain a population of NPCs. These NPCs were further maintained under conditions optimized for adult neural progenitors, such as three-dimensional spheroids (neurospheres) in the presence of Fibroblast growth factor 2 (FGF2) and Epidermal growth factor (EGF). All these approaches eventually led to the formation of cells with neural phenotype. However, the population of cells was always heterogeneous, conditions undefined, and the production of NPCs inefficient and time-consuming. In contrast, new approaches were designed to make the process of neural differentiation well defined, robust, and possibly exploitable by regenerative medicine in the future [17–20].

The initiation of neural differentiation in iPSCs leads to a series of morphogenic events resulting in the formation of radially organized cellular structures called neural rosettes [18, 21–23]. These structures are regarded as an early stage of neural development in vitro. Resembling the neural tube, neural rosettes function as a reservoir of NSCs. At this stage, NSCs can be isolated and further propagated in vitro without losing their characteristics. They can also be directed to differentiate into both neuronal and glial cell types [18]. Their constancy in differentiation potential and self-renewing capacity during in vitro cultivation has been evaluated in depth [19, 24].

The majority of protocols that have been established to generate NSCs rely on the formation of neural rosettes as a source of NSCs [17–20, 25, 26]. Initially, the typical approach builds on the growth of 3D cellular structures called embryoid bodies (EB). EBs are left to differentiate in a defined medium with FGF2 until neural rosettes appear or eventually become a predominant and distinguishable part of the EB [20]. In adherent conditions, a highly reliable protocol to generate NSCs introduces dual inhibition of *SMAD* signaling in iPSCs as the initial step ([25], See BOX 2 for further details). In this protocol, the inhibition of SMAD by both Noggin and SB431542 together with a specific initial density of cells leads to successful neural differentiation and formation of neural rosettes within 11 days [25]. This method generates a high number of *PAX6* + neural cells competent of rosette formation in as short as 11 days. However, in most cases, the neural conversion of iPSCs yields heterogeneous populations of NSCs and other neural cell types. Therefore, the isolation of NSCs from their niche remains a challenging and critical step in their further propagation. So far, few approaches have been introduced to address this problem. Selective enzymatic digestion has been used to separate neural rosettes and NSCs from EBs [20]. Another approach achieved the enrichment of the population of NSCs by fluorescent activated cell sorting (FACS) based on the characteristic combination of cell-surface markers [27, 28]. Interestingly, a different combination of these surface markers can be used to sort neurons or glia. Other protocols reached the homogenous population of NSCs by manually picking and re-plating neural rosettes until a colony of morphologically identified NSCs was established and expanded [17, 19, 24]

Besides the protocols that recapitulate the developmental processes of differentiation, direct conversion of somatic cell types into induced NSCs (iNSCs) has also been explored to gain self-renewing populations of NSCs. The initial reprogramming of fibroblast into iNSCs was achieved using transient expression of four reprogramming factors (*OCT4*, *SOX2*, *KLF4*, *C-MYC*) in mouse embryonic fibroblasts under neural inductive conditions [29] or by using just three of these factors (*SOX2, KLF4,* and *C-MYC*) while limiting the expression of *OCT4* [30]. Another study described a set of nine transcription factors (*SOX2*, *KLF4, C-MYC*, *BRN2*, *ASCL1*, *NGN2*, *HES1*, *ID1*, *PAX6*) to be efficient in converting mouse fibroblasts and Sertoli cells into iNSCs [31]. Eventually, the list of transcription factors narrowed to three (*SOX2*, *FOXG1*, and *BRN2),* or to *SOX2* alone, respectively [32, 33], which have been demonstrated to assure the derivation of iNSCs from both mouse and human fibroblasts. Additionally, studies on human cord blood-derived CD133 + cells confirmed that overexpression of *SOX2* alone, or in combination with *C-MYC* leads to the derivation of neuronal cells [34]. A small fraction of these cells then represented self-renewing neural progenitors. Lastly, two direct conversion protocols for iNSCs from peripheral blood mononuclear cells were recently reported [35, 36]. Both approaches are based on transient overexpression of two (*SOX2, C-MYC*) or four (*OCT4, SOX2, KLF4,* and *C-MYC*) transcription factors in combination with a set of small molecule inhibitors, cytokines, and hypoxic conditions. Taken together, transcription factor SOX2 seems to be inevitable for the direct conversion of iNSCs and maintenance of their phenotype from both mouse and human somatic cells. However, for successful derivation of iNSCs, its overexpression should be always complemented by either other mentioned transcription factors, small molecules, cytokines, and/or hypoxic conditions.

BOX 2: BMP signaling and the “Dual SMAD” inhibition.

**Table:** 

BMP signaling pathway is one of the major morphogenic regulators of embryonic development. As part of the TGF-ß superfamily, the role of the BMP protein family in this development is extensive. Studies of neural induction in vivo have shown that inhibition of BMP signaling is critical for the ectoderm to initiate the program of neural development. The binding of BMPs (especially BMP4) to the BMP receptor leads to the phosphorylation of SMAD1,5,9 proteins. Phosphorylated SMAD protein associates with SMAD4 and, translocated to the nucleus, acts as a transcription factor for genes driving epidermal differentiating programs. However, activation of TGFβ receptors leads to the phosphorylation of SMAD2,3 and their binding to SMAD4. In the nucleus, this complex induces mesodermal gene expression (reviewed in [13]). In the so-called “Dual SMAD” inhibition protocol, which is widely used to induce neural differentiation of iPSCs, both BMP and TGFβ signaling are inhibited simultaneously. Inhibitors and small molecules such as NOGGIN or LDN193189, and SB431542, respectively, drive the differentiation of iPSCs specifically towards neuroectoderm [25]	

### Neurons

In the context of neurodegenerative diseases, neurons are naturally the primary interest of the main research focus. Thus, to generate neurons in vitro, several methods can be employed depending on the experimental question that needs to be answered. The most straightforward approaches implement the fact that isolated neural rosettes or NSCs/NPCs will, under non-self-renewing conditions, differentiate towards neurons spontaneously [17, 19, 22]. Therefore, either isolated rosettes or NSCs/NPCs are kept in the cell culture media without growth factors such as FGF2 or EGF. This strategy can be modified by adding specific growth factors/small molecules to enhance the speed of the differentiation towards neurons or to direct the differentiation towards specific neuronal subtypes [17, 19, 37] (See BOX 3 for further details). Usually, factors such as brain-derived neurotrophic factor (BDNF), glial cell line-derived neurotrophic factor (GDNF), cyclic adenosine monophosphate (cAMP), Forskolin, or RA are used. Additionally, a cocktail of small molecules that activate or inhibit developmental signaling pathways, such as dual SMAD inhibitors with Sonic hedgehog (*SHH*) and *WNT* signaling activators, are used in the case of forebrain dopamine neurons [38, 39]. It is also of note that the intrinsic level of Wnt signaling in iPSCs significantly influences the spatial and regional axes of neuronal development in vitro*,* and the effects of signaling differences can be rescued by exogenous pathway activation [40]. However, these differentiation methods all generate a mixed population of neurons and glia in a relatively lengthy process of at least 60 days in in vitro culture. For the neurons to become really mature and electrophysiologically active, the period of differentiation could be as long as > 100 days [41]. Additionally, if pure neuronal culture is necessary for the final analysis, subsequent isolation of mature neurons via selection methods must be employed. Such selection methods could include FACS sorting [28] or selective isolation based on regulated cellular adhesion and suppression of growth of proliferating glial cells by AraC [42].

Additionally, some protocols directly stimulate iPSCs to form neurons without going through the stage of rosettes/NSCs/NPCs. These either use small molecule inhibitors that, when added sequentially every (other) day to the cell culture medium, will predominantly transform the iPSCs towards a specific type of neurons within 16–20 days [43]. Alternatively, the transient overexpression of specific transcription factors became widely used to generate so-called “induced neurons” (iNs). The first successful generation of iNs from iPSCs was performed by forced expression of *BRN2*, *ASCL1*, and *MYT1L* transcription factors [44]. Eventually, it has been shown that overexpression of *NGN2* alone is sufficient to generate iNs that were morphologically mature in two weeks [45]. Following this research, the repertoire of protocols describing differentiation into various subtypes of neurons has widened. For example, forced expression of *ASCL* and *DLX2* transcription factors in iPSCs has been shown to lead to the production of GABAergic iNs [46, 47]. A combination of both approaches, such as programming with transcription factor *NGN2* and inhibition of *SMAD* and *SHH*, generated functional glutamatergic neurons [48].

The strategy of iNs, as described by Zhang and colleagues, has recently developed into a robust protocol that drives the differentiation of iPSCs into cortical neurons, referred to as the “i3N—i3neuron system” (i3 standing for integrated, inducible, isogenic). Based on the study of Zhang et al., where it has been shown that lentivirus-mediated expression of a single transcription factor *NGN2* is sufficient to induce rapid neurodifferentiation of iPSCs, a study by Wang et al. introduced an improved strategy where *NGN2* transgene was stably integrated under doxycycline-inducible promoter into a safe-harbor locus in iPSCs. After 3–4 weeks of differentiation, generated i3Ns were mature and physiologically active when co-culturing with glia [49].

With all these available protocols, it is essential to adequately consider the research question asked as the differentiation method can influence the study results. For example, if the development-related question needs to be answered, it is more appropriate to let the neurons differentiate spontaneously without specific growth factors. These growth factors/small molecules could help neurons overcome possible problems that they would have if they were differentiating spontaneously. On the other hand, if the analysis requires a pure population of neurons or if the study is only done on the terminally differentiated neuronal population, then the homogenous population of sorted or induced mature neurons is likely the adequate model to use. However, it is essential to note that the absence of glia in such a model system might influence the experimental results if the studied mechanism also affects other cell types.

BOX 3 Small molecules and transcription factors in neuronal differentiation in vitro.

**Table:** 

The most commonly used factors for neuronal differentiation in vitro are BDNF, GDNF, cAMP, Forskolin, and RA, all of which execute their functions via different mechanisms. BDNF binds TrkB receptor kinase and promotes the differentiation of progenitor cells into neurons [50]. GDNF acts as an activator of the ERK-1/2 and P13K/AKT pathways to support the survival of neurons [51]. cAMP activates CREB-mediated gene transcription associated with the dendritic length and the morphological maturity of the young neurons in a level-dependent manner [52]. The cellular level of cAMP can be raised by Forskolin, which activates the cAMP pathway [53]. RA, a powerful morphogene, activates RA receptor signaling and acts as an essential regulator in neural induction, proliferation, and differentiation [54]. Expression of BRN2, ASCL1, MYT1L transcription factors establishes and specifies the neural identity. Overexpression of the key transcription factor NGN2 rapidly affects complex mRNA and miRNA expression profile and mediates regulatory network mediating neurogenesis from stem cells in days [55]	

### Astrocytes

Astrocytes are the largest and most abundant glial cells within the human brain. Astrocytes were initially viewed as a predominantly supportive modulator of brain processes that engage in fundamental homeostatic processes, specifically in trophic, metabolic, protective, and detoxification functions. Specifically, these include the production of antioxidants, maintenance of the blood–brain barrier, synapse modulation, cytokine release, and metabolism of neurotransmitters, especially GABA and glutamate (reviewed in [56]). In contrast to these essential roles promoting neuronal functionality, reactive states of astrocytes induced upon cellular injury were repeatedly reported as toxic for neurons. Indeed, evidence of early/late astrocytic reactivity has been repeatedly reported in numerous neurodegenerative diseases (review in [57]).

Over the last ten years, various protocols have been developed to differentiate iPSCs into astrocytes [41, 58–61]. Current iPSC-based methods for the differentiation of astrocytes typically rely on either NSCs/NPCs [61–65] or the oligodendrocyte progenitor cell [66] intermediates to form astrocytes via a cocktail of growth factors and small molecules (See BOX 4 for further details). These iPSC-differentiated astrocytes were shown to be functional also for cell-based models of neuropsychiatric disorders in vitro [61, 62, 64, 65] or engraftment in vivo [62, 63, 66, 67]. Naturally, existing methods are slow (up to 6 months) [61, 63, 66] or require sorting to reduce heterogeneity [28, 68]. To overcome this complication, Tcw et al. (2017) identified a faster 30-day differentiation protocol adequate for the assays for a neuroinflammatory response, phagocytosis, and spontaneous calcium activity [41]. Additionally, protocols for induced astrocytes (iA) have also been published. Developed methods efficiently generate astrocytes in 4–7 weeks using the inducible expression of *NFIA* or *NFIA* and *SOX10* in iPSCs [69–71].

Importantly, after the derivation of astrocytes from iPSCs, several studies also aimed to prove that they are functional and active correspondingly to their counterparts in vivo, thus usable for neurodegenerative disease modeling. Santos and co-workers compared a specific response to interleukin 1β (IL-1β) and tumor necrosis factor-alpha (TNF-α) between iPSCs-derived astrocytes and human primary astrocytes. Both evoked pro-inflammatory responses with similar gene expression changes [72]. Furthermore, iPSCs derived astrocytes were also able to sequester Aβ-plaques, exhibit altered Ca^2+^ homeostasis [73], display defective lipid metabolism [74], or switch into reactive astrocytes [72, 75].

BOX 4 Astrocytic differentiation and NFIA/SOX9.

**Table:** 

Astrocytes can be differentiated from iPSCs, NSCs, or OPCs by exposure to a set of mitogens and morphogens such as Ciliary neurotrophic factor (CTNF), BMP, FGF2, Leukemia inhibitory factor (LIF), and Fetal bovine serum (FBS) in a manner mimicking physiological developmental stages [41, 76]. Canonically, CNTF, FGF2, and LIF activate the JAK/STAT pathways and BMPs signal primarily through SMAD pathways, eventually converging in the regulation of GFAP expression [77, 78]. Additionally, direct reprogramming of astrocytes is based on a transient expression of a transcription factor NFIA [71]. The acquisition of glial competency is associated with the lengthening or arrest of the G1 phase upon upregulation of *CDKN1A* in high NFIA levels. Similar results have been obtained by upregulation of NFIB and SOX9 [69]	

### Oligodendrocytes

During development, oligodendrocyte progenitor cells (OPCs) give rise to mature oligodendrocytes, both of which are found in the mature CNS [79]. The key function of oligodendrocytes is to produce myelin and thus assure neuronal connectivity and axonal protection. Importantly, demyelination of neurons is often seen in neurodegenerative diseases [80].

The first protocol to describe the directed differentiation of oligodendrocytes from iPSCs was introduced by Nistor et al. in 2005 [81]. They used a combination of neurosphere formation followed by culturing cells in “glial restriction medium” and manual selection of OPCs. Their transplantation to shiverer mice resulted in integration, oligodendrocyte differentiation, and compact myelin formation, demonstrating that these cells display a functional phenotype [81]. Indeed, this protocol later led to the first human clinical trials of human embryonic stem cells (hESCs) derived OPCs for the treatment of acute spinal cord injury. Later, Izrael et al. (2007) differentiated oligodendrocyte progenitors from hESCs by firstly inducing the level of BMP by RA and its subsequent inhibition by NOGGIN. These cells were able to myelinate axons in mice brains as well as differentiate into mature oligodendrocytes [82]. Notably, Hsieh et al. (2004) published an important finding that Insulin growth factor-I (IGF-I) stimulates the differentiation of multipotent adult rat hippocampus-derived neural progenitor cells into oligodendrocytes [83]. This finding then stimulated the use of IGF-I in the differentiation protocols for iPSCs (See BOX 5 for further details) [84–88].

However, these initial protocols based on the recapitulation of the neural development of hESCs or iPSCs using growth factors alternation (such as RA, EGF, FGF2, SHH, or platelet-derived growth factor (PDGF)), cell culture adaptations with regards to extracellular matrix protein composition, and usage of small molecules like dual SMAD inhibitors, and ROCK inhibitor, have been dealing either with low yields or an exceptionally long differentiation procedure. New protocols, therefore, introduced shortened differentiation times due to forced expression of transcription factors. Notably, SRY-Box Transcription Factor 10 (*SOX10)* and Oligodendrocyte transcription factor (*OLIG2)* were identified as superior in oligodendrocyte cell-fate specification. Therefore their combination was used in protocols describing differentiation of iPSCs and NPCs into first oligodendrocyte progenitor cells followed by maturation into oligodendrocytes [46, 89–92]. Later, the overexpression of stably integrated single transcription factor *SOX10* was demonstrated sufficient to convert iPSCs into myelinating oligodendrocytes in only 22 days [93]. Additionally, a combination of IGF-I with promyelinating drugs ketoconazole and clemastine has been used to promote oligodendrogenesis in 3D cortical spheroids, offering another approach to the generation of oligodendrocytes in complex differentiation strategies [94].

BOX 5: Pathways involved in oligodendrocyte differentiation.

**Table:** 

Oligodendrocyte derivation often involves the generation of OPCs that serve as common precursors to both oligodendrocytes and astrocytes. OPCs can be produced from iPSCs through the use of various mitogens (e.g., FGF2, PDGF, and EGF) [95–98] and morphogens (like RA) or small molecules that promote SHH signaling (e.g., Smoothened agonist) or repress WNT/β-catenin signaling [99, 100]. Oligodendrocyte maturation from OPCs is often facilitated using IGF1 and triiodothyronine (T3). IGF1 seems to act through inhibition of BMP signaling (which induces maturation of OPCs into astrocytes) or through activation of ERK1/2 kinases [83, 101]. On the other hand, T3 was shown to upregulate transcription factor KLF9, which is likely involved in oligodendrocyte maturation [102]. Additionally, promyelinating drugs clemastine and ketoconazole inhibit enzymes for cholesterol biosynthesis, leading to the accumulation of sterol intermediates that promote maturation of OPCs into oligodendrocytes [94, 103] Direct differentiation of NPCs into oligodendrocytes is achieved via overexpression of transcription factors such as SOX10, OLIG2, and NKX6.2 [104]. These directly or indirectly affect the expression of genes involved in oligodendrocyte differentiation, such as PDGF receptor alpha (PDGFRA) or negative regulator of hedgehog signaling SUFU [105–108]. SOX10 was identified as a key transcription factor that was successfully used alone for oligodendrocyte differentiation. However, care must be taken when selecting the protocol for NPC generation as it may potentially affect the success rate of oligodendrocyte differentiation using SOX10 only [93]	

### Microglia

Microglial cells are part of the innate immune system and represent the mesoderm-derived cell type present in the human brain. Specifically, microglial cells originate from c-Myb-independent primitive macrophages present in embryonic yolk-sack. These primitive macrophages then migrate through embryonic vasculature, finally reaching primitive neuroepithelium and subsequently colonizing developing brain parenchyma [109]. They play numerous vital roles in brain development, homeostasis, and regulation of neuroinflammation [110].

The first microglia differentiation protocol was not presented until 2016 by Muffat et al. [111]. Since then, many protocols have been introduced and recently reviewed (See BOX 6 for further details) [112]. Interestingly, two strategies to gain microglia arose out of the published protocols. The first relies on the formation of yolk sack EBs, which are further differentiated into microglia-like precursor cells and microglia-like cells [111, 113]. Another differentiation scheme produces microglia-like cells through the stage of hematopoietic precursors [114, 115]. In these protocols, a cocktail of growth factors including BMP4, FGF2, vascular endothelial growth factor A (VEGF-A) together with different types of interleukins is supplemented to direct the differentiation. Also, co-culture with supportive cell lines, either astrocytes [116] or neurons [117], which provide the cells with essential factors, represent another possible and faster approach in deriving a mixed population of microglia-like cells.

It is of note that the results of in vitro differentiation into microglia are referred to as “microglia-like” cells since broad consensus on phenotypic and genotypic markers of these cells regarding their in vivo counterparts has not yet been fully established.

BOX 6 Inducible microglia.

**Table:** 

Differentiation towards microglia in vitro is achieved by the addition of growth factors and interleukins such as Stem cell factor (SCF), Cell survival factor (CSF), and Interleukin 34 (IL34) (reviewed in [112]). Microglia are in vivo derived from precursor cells localized in the yolk-sac through a process dependent on PU.1 and interferon regulatory factor 8 (IRF8) [118]. Those two transcription factors were shown to be upstream of CSF-receptor (CSFR) signaling [119–122], which is the critical pathway for microglia establishment as the CSFR ^−/−^ mice entirely lack the microglia cells [123]. CSFR signaling can be induced by its primary activators CSF and IL34 [123]. Further promotion of microglia proliferation, migration, and phagocytosis is supported by SCF [124, 125]	

### Transdifferentiation Strategies

Over the last years, the accuracy of iPSCs-derived cellular models of neurodegenerative diseases has been questioned. This concern was mainly because the generation of iPSCs from somatic cells is accompanied by the juvenilization of these cells into an embryonic-like state. This juvenilization can be traced in epigenetic modifications, telomerase length, and other aspects, including mitochondria condition [126–128]. Therefore, the aging phenotype of the differentiated cells derived from iPSCs seems not to correspond to their in vivo counterparts. Even though these properties make iPSCs-derived neural cell types great candidates for transplant therapies, their ability to recapitulate pathological features of age-associated neurodegenerative diseases with late-onset in vitro is considered limited (reviewed in [129–131]).

As a possible strategy to bypass this limitation, overexpression of progerin, a truncated form of lamin A associated with premature aging, has been shown to trigger neuronal aging phenotypes in iPSCs-derived neurons [132]. Furthermore, telomerase inhibition in iPSCs and subsequent neural differentiation has effectively shortened telomeres and thus provoked age-related phenotype in dopamine neurons [133]. Additionally, the application of chemical factors resulted in increased stress and, by consequence, the aging phenotype in cultured neuronal cells. Notably, in some cases, only after additional aging promoting elements were added was the studied neurodegenerative disease fully manifested [134].

A different way to preserve age-associated features in cells is the transdifferentiation of somatic cells directly to the desired cell type (reviewed in [130, 131, 135]). This method has caught emerging attention in neurodegenerative disease modeling. The first conversion of human fibroblasts has been carried out by overexpression of *BRN2*, *ASCL1*, *MYT1L*, and *NEUROD1* [44]. These factors have also been used to reprogram human hepatocytes into iNs [136]. Since then, protocols have introduced alternative combinations of reprogramming transcription factors and microRNAs to direct somatic cells into neuronal lineage [137–139]. So far, many protocols to generate dopaminergic, glutamatergic, cholinergic, GABAergic, and other neurons have been developed (reviewed in [130]). Importantly, avoiding the iPSCs stage, transdifferentiated iNs were shown to conserve the age-related epigenetic landscape and other cellular properties from the cell of origin [138, 140]. Therefore, deriving aged neural cell types adequately without the need to induce aging in vitro is undoubtedly beneficial for neurodegenerative disease modeling. However, recent work shows extensive de novo DNA methylation occurs in mouse fibroblasts directly converted to neurons using BAM factors [141]. While this study suggests that this epigenetic remodeling promotes a neuronal epigenetic landscape, a more detailed analysis of epigenome remodeling that may occur in directly converted human neurons in aged or disease states is warranted.

### 3D Brain Organoids

Cerebral organoids are iPSCs derived self-assembled 3D cellular aggregates mimicking human fetal brain development [7, 142]. They display multiple relevant cell types that undergo intrinsic developmental patterns of a particular modeled organ. Additionally, an organoid’s ability to obtain specialized functions ordinarily present in a modeled organ proved to be a novel tool for studying the human brain. A 3D cerebral organoid organ-like organization can be subsequently used to investigate human fetal brain development, tissue organization, aging, metabolic processes, drug screening, and disease modeling (reviewed by [143]).

Derivation of human cerebral organoids nowadays relies on both guided and unguided methods (reviewed by [144]). Initially, protocols were dependent on stem cells’ intrinsic ability to assemble and differentiate towards the neuronal fate. This approach led to the formation of whole-brain 3D organoids with different regions within the organoid [142]. Subsequent protocols modified the original procedure to form region-specific cerebral organoids. Those “guided” organoids are directed by region-specific growth factors, differentiation factors, and specific cellular inhibitors. For this reason, region-specific cerebral organoids are more representative of cellular composition, structural features, and molecular processes of particular brain regions modeled [144]. Since then, region-specific organoids such as cortical spheroids [145], cerebellar organoids [146], hippocampal organoids [147], and midbrain organoids [148] were established, contributing to our understanding of brain region specificities.

Notably, refinements of 3D cerebral organoid cultures opened new possibilities for investigating human brain disorders in vitro and possibly overcoming the gap between in vitro human cell-based systems and animal models. Indeed, animal models often fail to reproduce human-specific pathology. However, even 2D human cellular cultures are misrepresentative of complexly interacting in vivo environments. Therefore, the intricate 3D organization of cerebral organoids, especially considering the extracellular deposition of pathological proteins in vast neurodegenerative diseases, proves to open new research possibilities. Another well-addressed advantage of cerebral organoids is their potential to establish patient-derived models with the principal characteristic of a patient’s genetic information. Indeed, first patient-derived 3D cerebral are starting to be used, for instance, in drug-screening (reviewed by [149]) and glioblastoma research [150], thus opening new possibilities in personalized medicine. Despite current advancements in cellular biology, several limitations (e.g., heterogeneity, lack of vascularization, aging) need to be addressed to match all principal aspects of human brain physiology. Despite these limitations, cerebral organoids hold the promising potential to establish fresh new insights into neurodegenerative disease modeling.

## Alzheimer’s Disease

Alzheimer’s disease (AD) is a chronic neurodegenerative disease characterized by loss of neurons in the cerebral cortex and subsequent cortical dysfunction. It is the most common form of dementia, with 60–80% of all dementia cases significantly contributing to morbidity/mortality rates in the elderly population worldwide. There are two forms of AD—familial (fAD; 5%) and sporadic (sAD; 95%). The vast majority of fAD is associated with mutations in the Amyloid Precursor Protein (*APP*), Presenilin 1 and 2 (*PSEN1* and *PSEN2*) genes [151]. However, since most AD cases are considered sporadic, sAD is presumably triggered by the interplay of genetic and environmental factors with unclear etiology [151]. One of the major risk factors associated with sAD is the *APOE4* allele, with almost 65–80% of AD patients. In contrast, the *APOE2* allele is considered a protective factor. Apart from *APOE*, genome-wide association studies have identified more than 20 AD risk genes, including *SORL1* or *TREM2*, and detailed studies of these in the field of iPSCs are beginning to emerge [152–154].

Clinically, AD-associated progressive memory loss is underlaid by two main pathological features present in the AD brain: 1) extracellular beta-amyloid plaques and 2) intraneuronal Tau-containing neurofibrillary tangles. Hallmarks of the first feature include the accumulation of insoluble deposits of amyloid β peptides (i.e., "Aβ plaques") cleaved from APP protein. APP is critical to neural stem cell development, neuronal survival, neurite outgrowth, and neuronal repair. It is, under physiological conditions, cleaved to short peptides by alpha, beta, and gamma secretases to perform its function. Gamma-secretase then consists of PSEN1 and PSEN2 subunits. Produced Aβ peptides are under physiological conditions predominantly 40-mers (Aβ40), but in the case of pathological mutations in APP or PSEN1/2 genes, the 42-residue peptides (Aβ42) are overrepresented [155]. These Aβ42 peptides have a markedly higher propensity to aggregate in comparison with Aβ40, causing the formation of dense, mostly insoluble deposits of Aβ plaques in the extracellular matrix. According to this “Amyloid hypothesis”, the deposition progressively leads to synaptic dysfunction, inflammation, neuronal loss, and, ultimately, dementia [156].

The second feature leading to AD pathology is “Neurofibrillary tangles”, insoluble aggregates of hyperphosphorylated microtubule-associated protein Tau. Tau protein is phosphorylated by a number of kinases (e.g., CDK5, GSK3, and others), and its abnormal phosphorylation promotes the polymerization and formation of insoluble filaments. Tangles then accumulate intracellularly within neuronal soma, resulting in the collapse of the axonal transport system [156]. However, the process of aggregation of hyperphosphorylated Tau (P-TAU) in the absence of causative mutation is unknown, and the exact relationship between P-TAU and Aβ has been elusive so far. Moreover, numerous clinical trials aiming to prevent P-TAU and/or Aβ deposition failed to demonstrate the effectiveness of disease-modifying treatments. This suggests that our understanding of the molecular basis of AD is incomplete and implies that protein aggregation is perhaps not a cause but rather a consequence of unknown mechanism(s) that eventually lead to AD pathology. Taken together, despite a rising number of publications and data, the exact pathophysiologic mechanisms underlying AD is still mostly unknown.

**Fig. 2:**
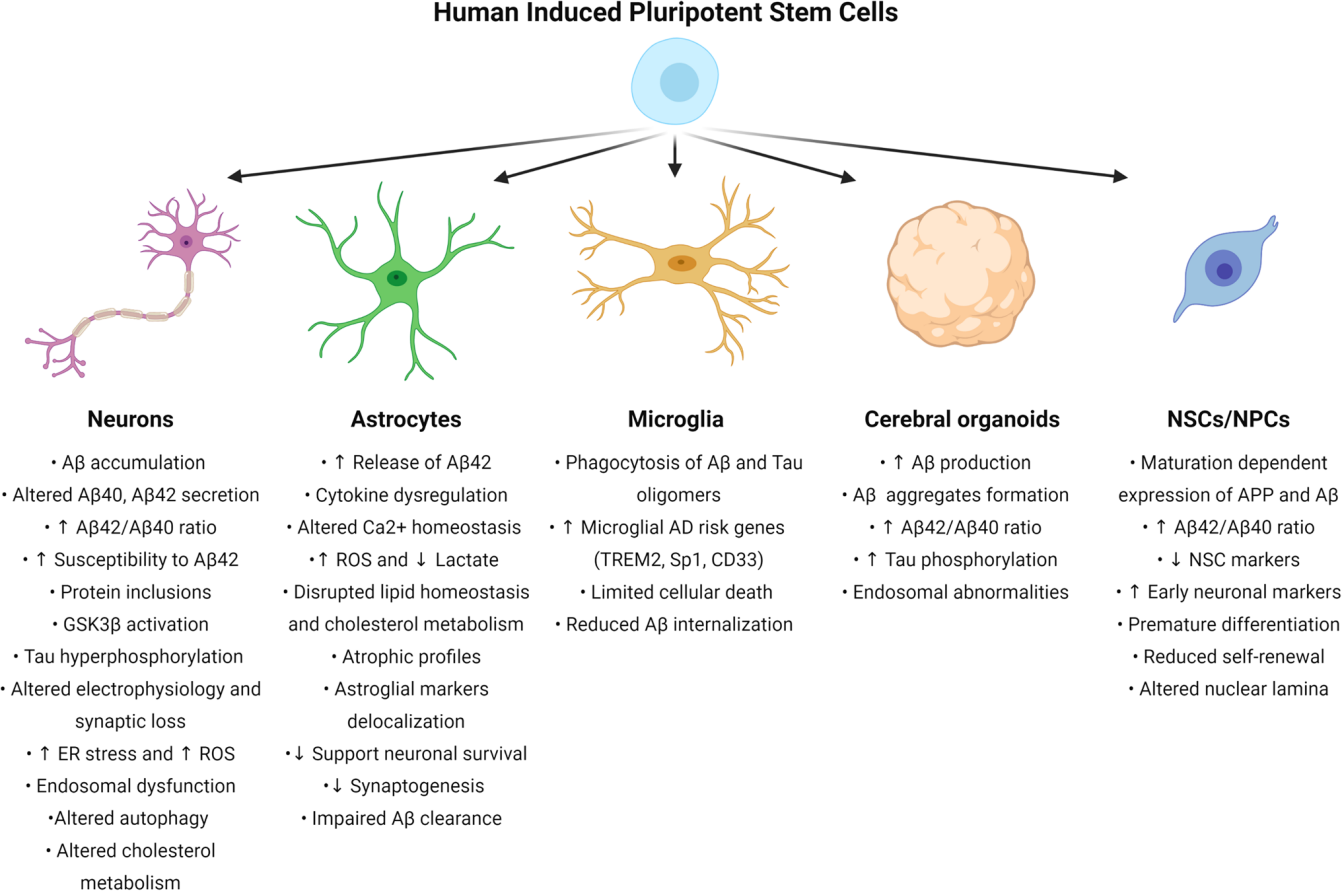
Major finding from stem-cell-based models of AD. For each cell type, we summarize significant results presented in Sect. 3. “↑” represents upregulation or increase, “↓” represents downregulation or decrease

The broad applicability of different AD-based iPSCs significantly contributed to the current understanding of AD. Still, an increasing number of AD-iPSCs in repositories from patients with sAD or fAD or genetically manipulated cell lines [158–160] in combination with precisely refined cellular differentiation protocols into neurons, all central glial cells and microglia represent an efficient method for examination of AD. This may not be limited to etiopathogenesis, pathophysiology, molecular pathology, and drug testing of Alzheimer’s disease. Here we provide an overview of major iPSCs-derived cellular AD models in vitro and summarize the significant findings from these models in Fig. 2. Additionally, we also encourage readers to explore other relevant reviews on this topic as they provide a different perspective on AD development and in vitro modeling [161–168].

### NSCs and Progenitor Cells

Although the majority of the AD-iPSC studies focused on studying neurons, there are, to this date, six reports that, at least to some extent, addressed the phenotype, behavior, and/or molecular changes coupled to AD specifically in NSC/NPCs [169–175]; see Table 1 for details). In the first study, Koch et al. [170] used lentiviral transgenesis and introduced fAD-causing mutations in *PSEN1* (*L166P*, *D385N*) to long-term self-renewing NSCs derived from hESCs. The study shows that the expression of APP and Aβ secretion is maturation-dependent. They detected very low levels of APP and Aβ in self-renewing NSCs while the expression of both proteins increased to detectable levels only after three weeks of neuronal differentiation in vitro. Upon introducing mutant *PSEN1* variants to NSCs, they showed no effect on apoptosis, but significant downregulation of proliferation compared to wild-type *PSEN1* transduced NSCs [170].

**Table 1:**
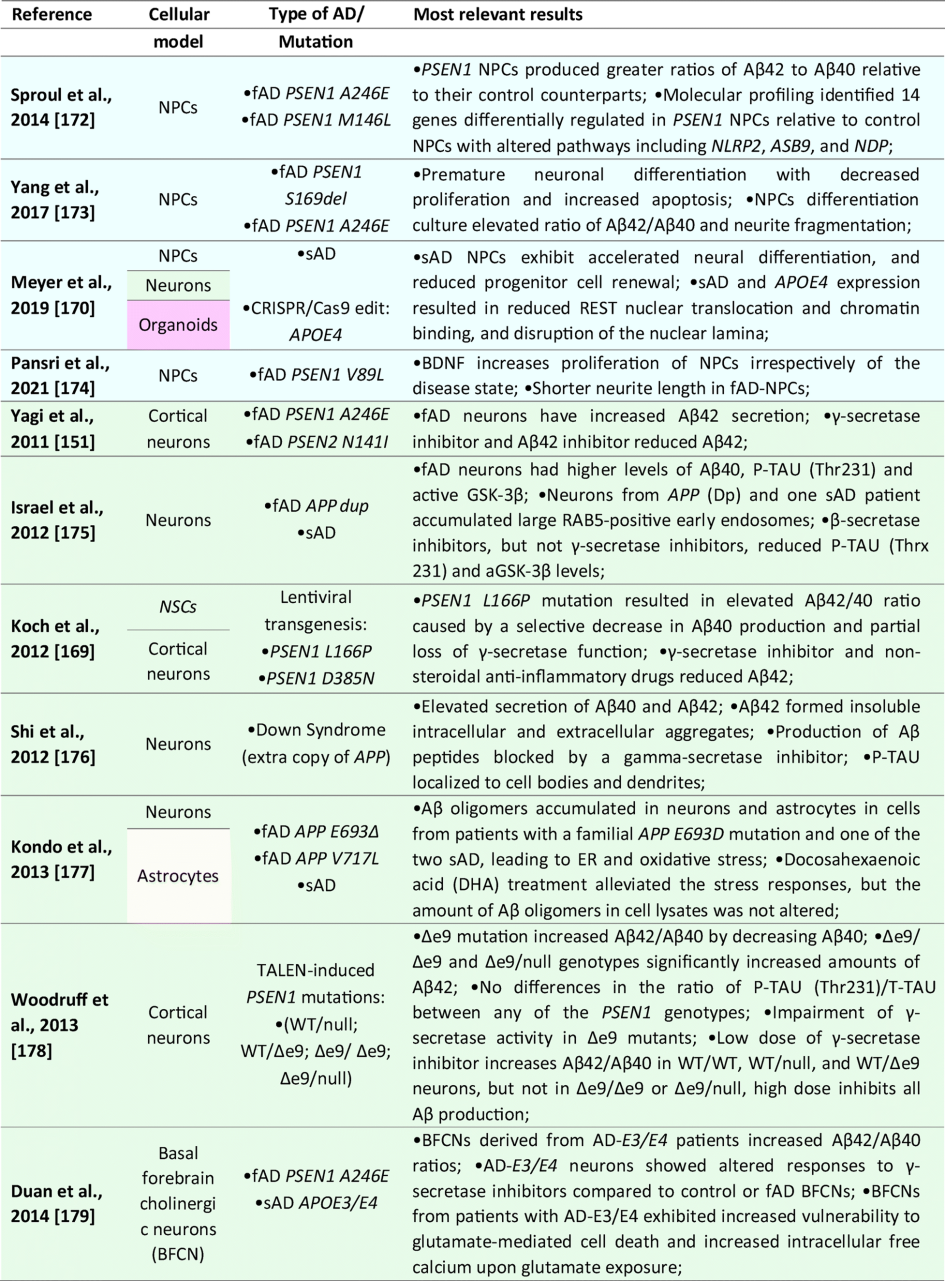

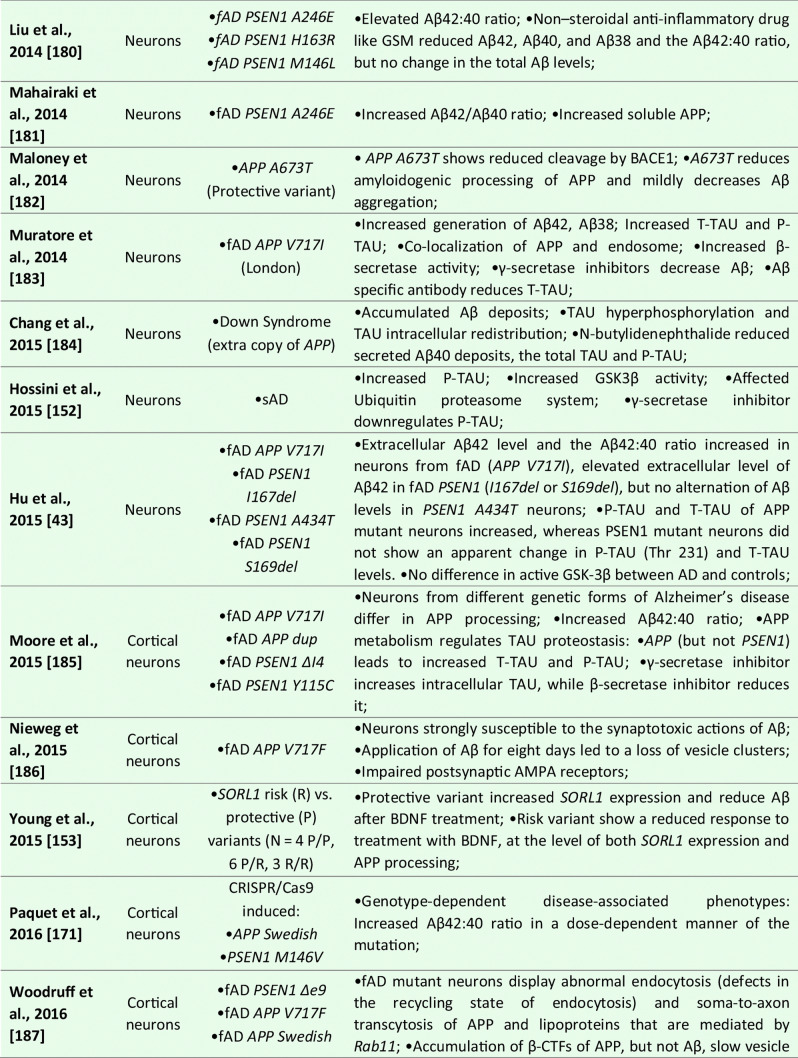

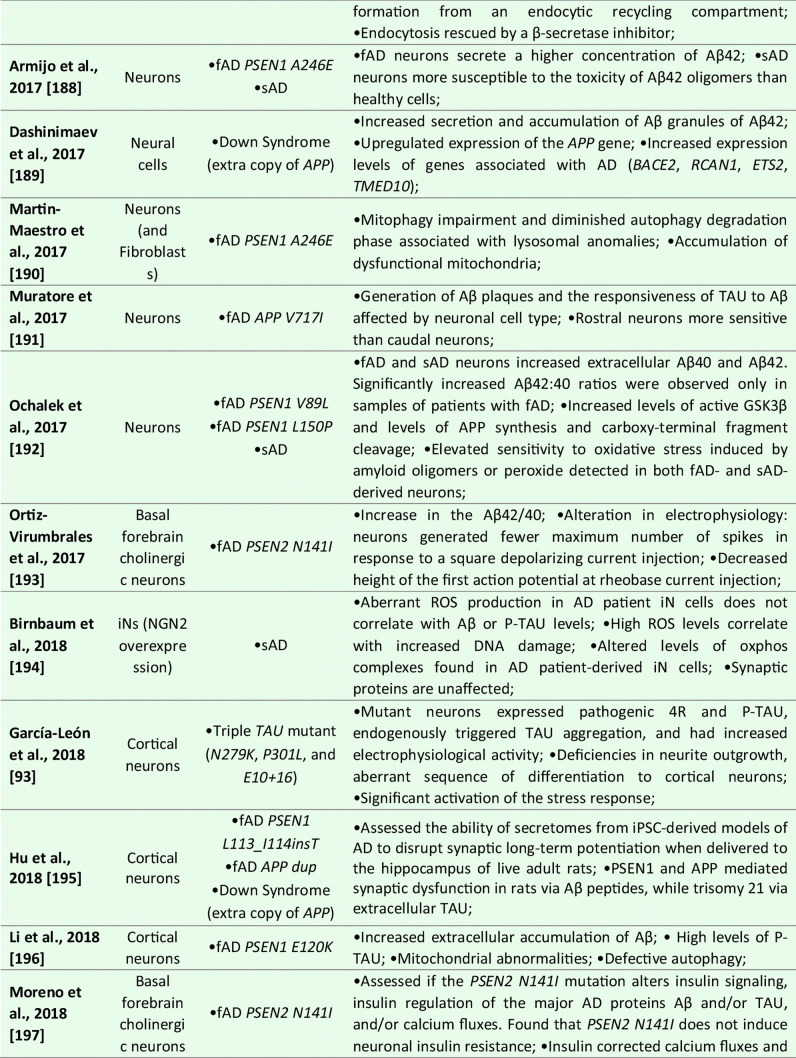

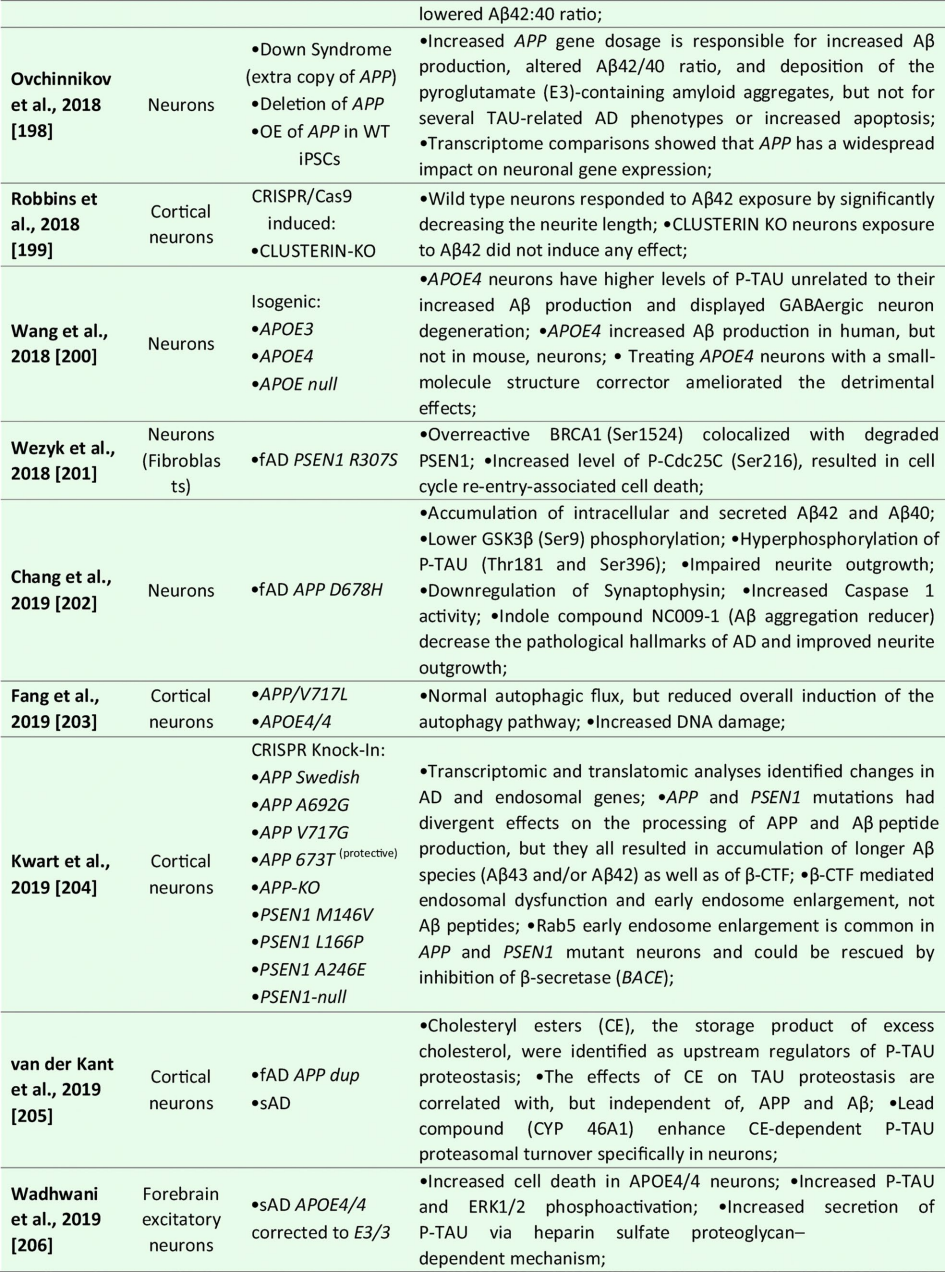

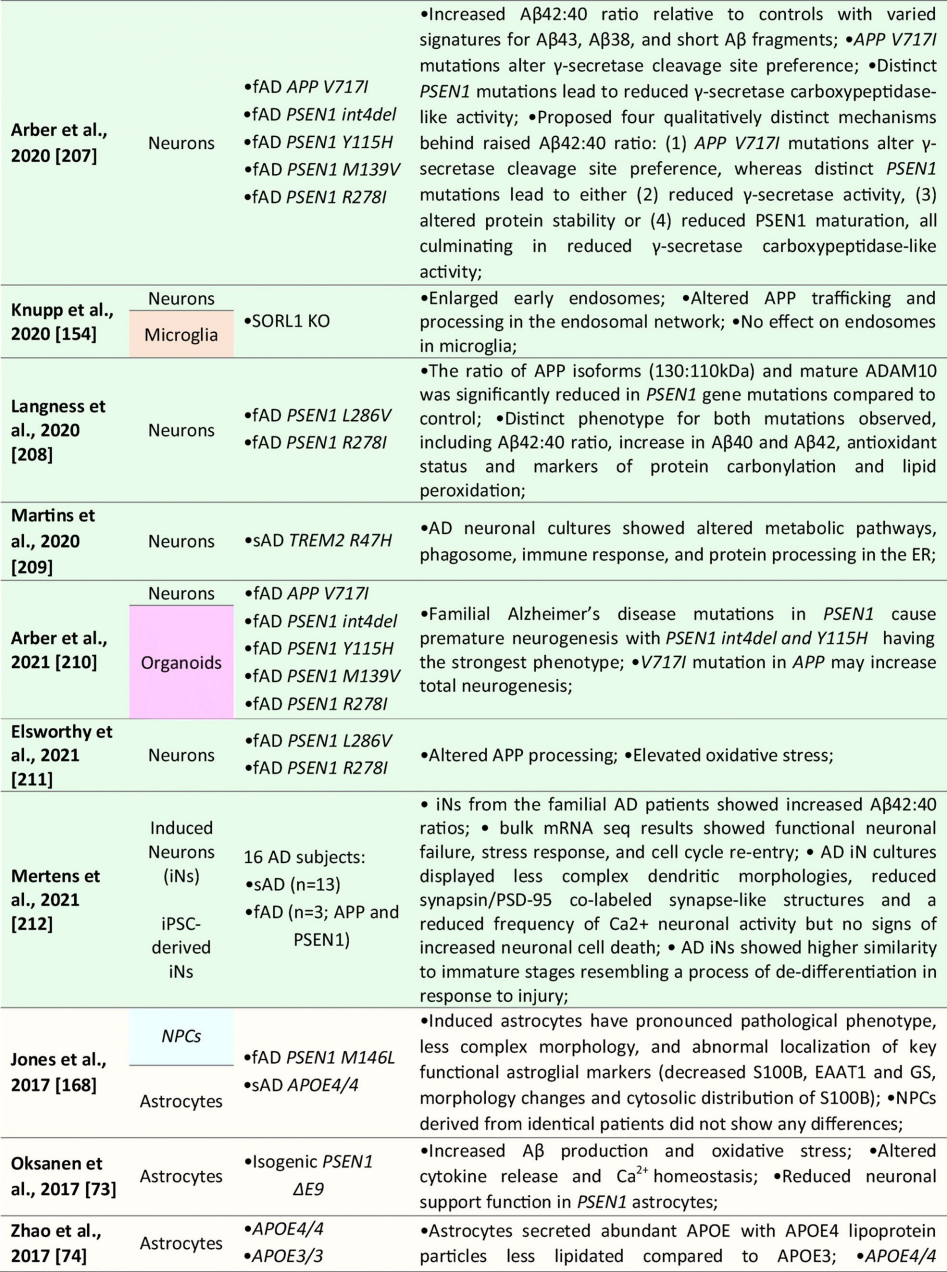

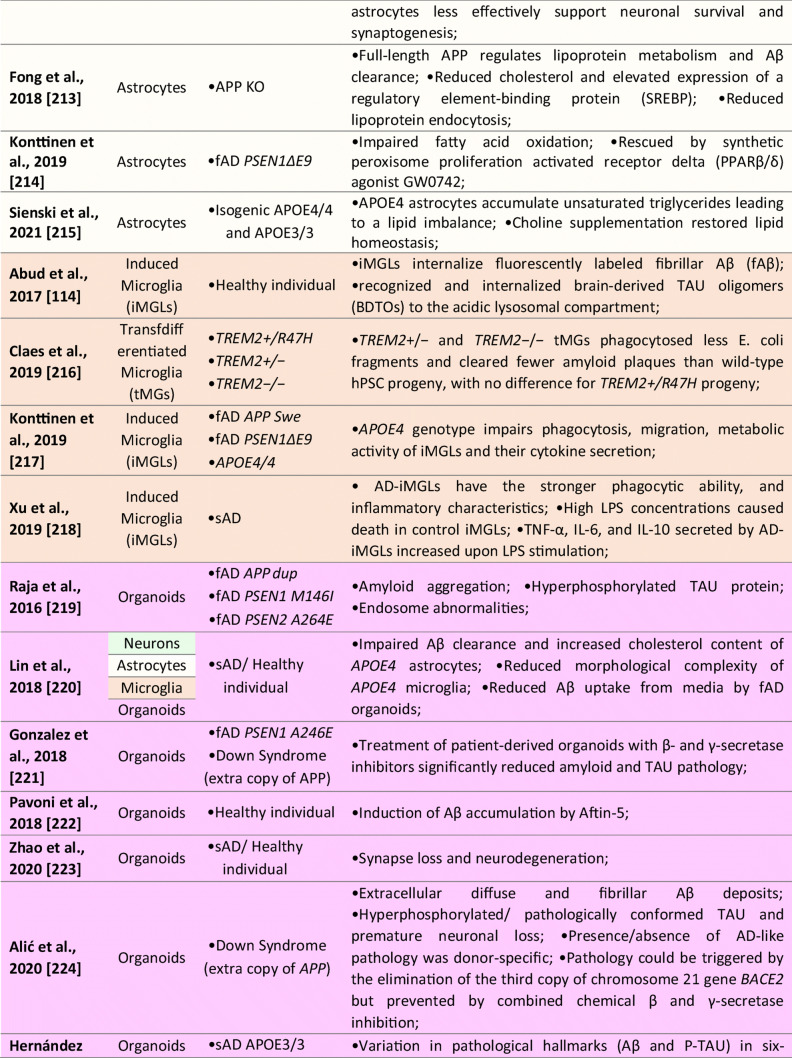

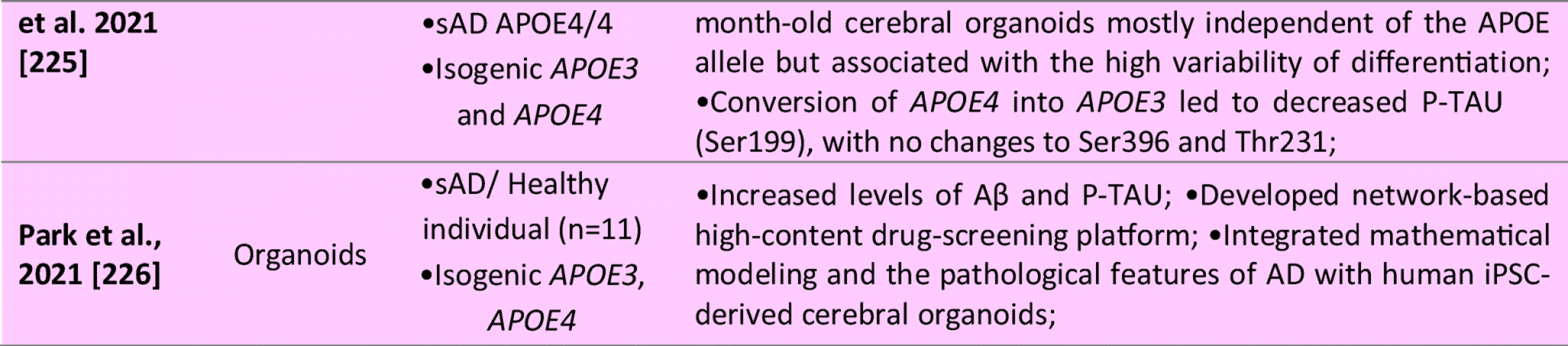
List of publications utilizing human iPSC-derived neural models for studying Alzheimer’s disease

However, the next two studies did not find any specific aberrations in NSCs that would be related to AD phenotype. Sproul et al. [173] generated iPSCs from affected and unaffected individuals from two families carrying *PSEN1* mutations (*A246E* and *M146L*). *PSEN1* mutant NPCs had greater ratios of Aβ42 to Aβ40 relative to their control counterparts but did not show any aberrations in proliferation. Molecular profiling identified only 14 genes differentially expressed in *PSEN1* NPCs relative to control NPCs, including *NLRP2*, *ASB9*, and *NDP* [173]. Later, Jones et al. [169] reported the generation of human iPSCs from healthy individuals and patients with either early-onset fAD (*PSEN1 M146L*) or the late-onset sAD (carrier of *APOE4/4*). They also report no significant differences in NPC growth rates or NPC marker expression (*PAX6* and *NESTIN*). No significant difference was detected in the efficiency of early neuronal induction (measured by the proportion of β-III-tubulin + neurons) between any individual. Both sAD and fAD NPCs retained their characteristic morphology, expression of canonical markers and were able to generate mature cortical neurons with the same efficiency as control NPCs [169].

Interestingly, a recently published (and perhaps the most detailed) study was performed by Meyer et al. [171]. They generated iPSCs from a larger cohort of sAD patients and age-matched controls. Gene expression analysis of sAD-NPCs showed a marked increase in the expression of neural differentiation-related genes (including *ASCL1*/*MASH1*, *DCX*, *MAPT*, *CD24*, and *STMN2*), premature neuronal differentiation (a finding supported by the study of Yang et al. [174]) and reduced NPCs self-renewal. Importantly, this phenomenon was not caused by the *APOE4/4* genotype as *APOE3/3* sAD cell lines showed the same trend [171]. Functional analysis of the transcriptome of sAD NPCs (and neurons) suggested that upregulated genes were regulated by the transcriptional repressor *REST* (repressor element 1-silencing transcription factor). Indeed, sAD NPCs showed reduced nuclear REST levels and REST-RE1 site binding. A similar differentiation phenotype and involvement of *REST* were observed in isogenic neural cells generated from iPSCs that were gene-edited to express APOE4. Conversely, gene editing of *APOE4* to the neutral allele *APOE3* reversed the phenotype. Finally, they were able to show that the loss of function of *REST* in sAD and upon *APOE4* expression was due to reduced nuclear translocation and chromatin binding and was associated with disruption of the nuclear lamina. These findings suggest that *REST* dysfunction and epigenetic dysregulation emerge in sAD and *APOE4* NPCs and persist in differentiated neurons, potentially contributing to the onset of AD [171].

Taken together, it would seem that while some fAD causing mutations in *PSEN1* might not affect the properties of NPCs [169, 173], there are at least some fAD and sAD-patient’s derived NSCs that show a significant decrease in proliferation, downregulation of NSC-specific markers, upregulation of early neuronal markers and show signs of premature differentiation [170, 171, 174]. Meyer et al. further show that in sAD, this is due to the inactive *REST* complex and the disruption of the nuclear lamina. These findings raise the possibility that a developmental perturbation, such as the depletion of NSCs or altered neural circuit formation, may occur early in life in individuals predisposed to develop sAD. Moreover, while this may not significantly compromise cognitive function in young adults, it may increase the risk of neurodegeneration and cognitive decline when combined with chronic stressors later in life [171].

### Neuronal Models

To this date, over 50 studies were published that focused on studying AD and AD-related phenotypes using iPSC-derived neurons (See Table 1 for details). These reports mainly focus on studying fAD mutations in *APP*, *PSEN1*, and *PSEN2* or employ the strategy of iPSCs generation from sAD patients, although CRISPR/Cas9 gene-edited cell lines are being used more and more often. Several studies then specifically focused on addressing the role of *APOE4* genotype, other risk factors (*SORL1* or *TREM2*), or mutant TAU on AD development. Methodologically, they employ a large variety of differentiation protocols for neuronal derivation ranging from glutamatergic [176], GABA-ergic [177], and dopaminergic subtypes to basal forebrain cholinergic neurons [178, 179], and several studies also use direct transdifferentiation, generating iNs [140, 180].

In general, initial studies using iPSCs-derived neurons demonstrated the presence of typical AD pathological features in these cultures. They described 1) Aβ accumulation in cell cultures; 2) altered secretion of Aβ40 and/or Aβ42 peptides; 3) the presence of protein inclusions; 4) activation of glycogen synthase kinase 3 beta (GSK3β); and 5) TAU hyperphosphorylation (summarized in Table 1). Subsequent studies validated that AD-iPSC-derived neurons can also demonstrate 6) loss of synapses and decreased synaptic plasticity [181, 182], 7) altered electrophysiological activity [93], 8) increased oxidative stress and reactive oxygen species (ROS) generation [183, 184], 9) endosomal dysfunction [185], 10) defective autophagy, mitophagy and mitochondrial abnormalities [186–188], and 11) altered cholesterol metabolism [189]. These pathologies were found both in fAD and sAD-iPSC-derived neurons, albeit not all fAD/sAD cell lines displayed all the pathological signs. One of the aspects possibly playing an important role in this phenomenon is the finding of Muratore et al., who reported that generation of Aβ plaques and the responsiveness of TAU to Aβ are affected by neuronal cell type with rostral neurons being more sensitive than caudal neurons [190]. Nevertheless, the application of β- or γ-secretase inhibitors (and possibly other small molecule inhibitors) in majority of the studies resulted in the reduction of Aβ peptides secretion and downregulation of P-TAU, thus confirming the possibility to use these in vitro models for drug discovery approaches [152, 170, 185, 191–196].

Numerous studies also investigated the effects of Aβ oligomers in AD iPSCs-derived neuronal cultures. In general, fAD neurons displayed a high Aβ42/Aβ40 ratio [73, 170, 172, 192, 193, 197]. Interestingly, increased Aβ oligomers have been shown to induce endoplasmic reticulum stress and ROS generation [198]. Indeed, increased oxidative stress and ROS-mediated cellular dysfunction were documented in both *PSEN1*^*A246E*^ [199] and mutant *APP*^*A693E*^ neurons [198]. Recently, Aβ42 oligomers were also reported to provoke mitochondrial DNA damage and decrease the effectiveness of DNA repair processes [183, 200]. Similarly, Ortiz-Virumbrales et al. observed altered electrophysiology of neuronal cells in case of an increased Aβ42/40 ratio [179]. Additionally, neuronal toxicity and disrupted functionality can also be obtained by exogenous Aβ administration [198, 201]. Notably, these exogenous Aβ oligomers preferentially induced toxicity in glutamatergic neurons compared to GABAergic neurons [202]. Lastly, studies also showed that fAD neurons (*PSEN1*^*A246E*^; *PSEN1*^*L150P*^) were more susceptible to Aβ42 than neurons derived from sAD patients or healthy controls [203, 204].

Interestingly, what emerges from recent studies of AD-iPSC-derived neurons is that the lipid and cholesterol metabolism and the intracellular trafficking defects may be an important common pathological process associated with AD and other neurodegenerative diseases [189, 205, 206]. A recent study by van der Kant showed that cholesteryl esters (CE), the storage product of excess cholesterol, are upstream regulators of P-TAU proteostasis and are independent of APP and Aβ [189]. Several other studies then investigated early endosomal-related defects in fAD [191, 193, 194], and a recent comprehensive iPSC-based study [185] suggests the presence of early endosome enlargement as a potentially unifying pathological hallmark of AD. Moreover, their RNA-seq analysis and ribosome profiling found multiple common endocytic/endosomal trafficking-associated genes dysregulated in all analyzed fAD mutant neurons (i.e., *APP-Swe*, *APP-A692G*, *APP-V717G*, *PSEN1-M146V*, *PSEN1-L166P*, *PSEN1-A246E*, *APP-KO*, and *PSEN1-null*). Many of these trafficking-related genes have been previously implicated in late-onset AD (e.g., *SORL1*, *CLU*, *APOE*, etc.). This, therefore, suggests that a shared network of cellular and molecular changes may underlie both sAD and fAD pathogenesis [185].

Additionally, it is of note that neurodifferentiation models based on pluripotent iPSCs and/or direct reprogramming techniques are beneficial to address the differentiation propensity of disease-relevant cell types. Curiously, experiments on AD neurons are now showing somewhat contradictory results. On the one hand, several robustly designed studies bring evidence that neurons derived from both sAD and fAD(PSEN1)-iPSCs differentiate prematurely [170, 171, 207]. On the contrary, a new publication by Mertens et al. very clearly shows that neurons induced directly from AD patient’s fibroblasts lack fully differentiated phenotype and have downregulated genes related to synaptic transmission, ion transport, and synaptic plasticity [180]. Thus, this study opens several interesting questions, including whether neuronal changes in AD result from the accumulation of damaging agents or rather a lack of a fully differentiated neuronal transcription. They also hypothesize that this “hypo-mature” state of AD-induced neurons might relate to the fundamental cell biological process of de-differentiation in response to injury [180].

### Astrocytes

In AD, astrocytes were shown to undergo initial atrophy with subsequent reactive astrocytic hypertrophy ([208] and reviewed by [209]). Both processes are thought to be accelerated by an astrocytic reaction to Aβ fragments in their vicinity [210, 211].

Several iPSCs derived fAD [73, 169, 212] and sAD [70, 74, 205] astrocyte models have been reported thus far (see Table 1 for details). In the case of fAD, Oksanen et al. demonstrated that fAD *PSEN1 E9* astrocytes show an increased release of Aβ42, cytokine profile dysregulation, altered Ca^2+^ homeostasis, increased ROS, and decreased lactate production [73]. Fong et al. reported that APP-KO astrocytes show reduced cholesterol levels and an elevated expression of the regulatory element-binding protein (SREBP), which are both downstream consequences of reduced lipoprotein endocytosis [212]. Jones et al. (2017) further showed that induced astrocytes derived from both sAD and fAD (*PSEN1 M146L*) patients exhibit a pronounced pathological phenotype. They showed significantly less complex morphological appearance, overall atrophic profiles, and abnormal localization of key functional astroglial markers (no glial fibrillary acidic protein (GFAP) changes but decreased S100B, EAAT1, and GS, morphology changes, and cytosolic distribution of S100B) [169]. Studies on sAD-iPSC-derived astrocytes additionally show *APOE4/4* astrocytes less effectively support neuronal survival and synaptogenesis and that the astrocytes secrete abundant APOE with APOE4 lipoprotein particles less lipidated compared to APOE3 [74]. Lastly, Lin et al. (2018) showed impaired Aβ clearance and increased cholesterol content of *APOE4* astrocytes [213], and a new study by Sienski et al. reveals disrupted intracellular lipid homeostasis in both astrocytes and microglia [205]. Overall, elucidating disease-specific cellular responses in astrocytopathies may be a crucial factor in AD progression and manifestation.

### Oligodendrocytes

Oligodendrocytes generate myelin sheaths around axons. However, it has been shown that a subset of proliferative, immature oligodendrocytes may play a role in neural repair [214]. Studies related to AD showed that the morphology of oligodendrocytes is altered in AD [215]. Additionally, Aβ oligomers caused a decrease in myelin proteins [216] and were toxic to oligodendrocytes [217]. And while oligodendrocytes have been successfully generated from iPSCs [104, 218, 219], the assessment of the role they may play in AD and their relevance in sAD models have, as yet, not been reported [166].

### Microglia

Neuroinflammation is implied as one of the defining features of neurodegenerative diseases. Under these pathological conditions, microglia adopt a reactive state with morphological and functional changes [220]. In AD, the first evidence of reactive microglia in neuritic plaques was described by Alois Alzheimer himself [221]. Reactive glial cells have been since documented in numerous other studies [222], where they cluster around Aβ plaques highlighting their inability to clear Aβ [223, 224]. Microglia are also implicated in the neuroinflammatory component of AD etiology, including cytokine/chemokine secretion, which worsens disease pathology [225]. The microglial reaction was considered only incidental and rather a functional response of microglia to the deposition of Aβ and neuritic plaques formation. However, recent genome-wide association studies identified several high-risk AD loci genes, namely *TREM2* [226], *Sp1*, *CD33* [227]. These genes are highly or even exclusively expressed in microglia, suggesting that microglial might be crucially involved in the initial causal pathogenesis of AD [228].

iPSCs-derived microglia (see Table 1 for details) manifested the ability to phagocyte AD-related substances, including β-amyloid and Tau oligomers [114]. Moreover, once exposed to fibrilar Aβ, microglial cells expressed different gene expression profiles with a predominant increase among microglial AD risk genes [114]. In another study, AD-iPSCs microglia displayed higher phagocytic ability with limited cellular death upon lipopolysaccharide exposure compared to wild-type microglia [229]. Recent studies also examined the role of the *APOE4* allele in microglial cells. Generation of *APOE4/4* microglial cells led to significant dysregulation of gene expression levels and reduced Aβ internalization [213]. Moreover, Lin et al. applied his concept of *APOE4/4* microglia and *APOE3/3* microglia to study the ability to internalize Aβ peptides from diffusely Aβ affected 3D organoids. *APOE3/3* microglial cells had higher activity of phagocytosis compared to *APOE4/4*. Comparatively to *APOE4*, additional studies with microglia-associated AD-risk factors, i.e., *TREM2* [230], were conducted to validate the feasibility of AD modeling using the iPSCs microglial models.

### 3D Models

While 2D cellular models of Alzheimer´s disease significantly expanded our understanding of AD, it has become apparent that more complex models will be needed to examine (1) the interaction between multiple cell types connected to AD pathogenesis; (2) complex 3D transcriptomics; (3) role of immune cells; (4) organization of neuronal populations; and (5) formation of neurofibrillary tangles and plaques in the complex tissue-like environment. This absence of cerebral complexity in 2D cell cultures has been recently overcome by developing 3D cerebral organoids from hESCs [142]. Cerebral organoids recapitulated with a remarkable degree of detail human CNS development and proved to be an excellent tool for disease modeling, including AD [171, 181, 207, 213, 231–236]. Initially, a 3D model of human neural stem cells overexpressing mutant PSEN1 and APP has been reported by Choi et al. [237]. This system was based on the culture of neural progenitor (ReN) cells in a thick layer of the extracellular matrix. Cells were genetically engineered to carry an AD-causing mutation in APP or PSEN1, and upon terminal differentiation, amyloid plaque-like and neurofibrillary tangle-like structures were observed. Functional studies showed that gamma-secretase inhibition reduced Aβ formation and led to a decrease in the P-Tau level [237].

Since this initial 3D model study of AD, ten publications described the generation of cerebral organoids from both fAD or sAD iPSCs and from the patient with Down syndrome (see Table 1 for details). These publications report increased Aβ production, formation of Aβ oligomer aggregates, increased Tau phosphorylation, synaptic loss, and endosomal abnormalities [171, 213, 231, 232, 234, 235]. Raja et al., as a first one, demonstrated that 3D AD cerebral organoids exhibit an increased Aβ42/Aβ40 ratio, which is considered one of the most specific biomarkers for AD. Following treatment of AD cerebral organoids by a γ-secretase inhibitor and β-secretase inhibitor significantly reduced the propensity of β-amyloid accumulation and Tau phosphorylation [234]. Interestingly, a new study by Arber et al. additionally reports that selected PSEN1 mutations may cause premature neurogenesis confirming their data from 2D neurons [207]. Additionally, another study utilized a small-molecule approach to induce β-amyloid aggregation posttranslationally by Aftin-5 treatment [233]. Here, the potential was extended to non-AD cell lines with Aβ shift towards Aβ42 production. Hence ideal for studying environmental factors contributing to AD etiopathogenesis.

Interestingly, a recent report shows evidence of innate microglia in developing cerebral organoids [238]. Following several publications embellishing the proof of a variety of macroglia that 3D cerebral organoids display [239, 240], a sophisticated approach in studying AD cellular interaction in 3D can be adapted. However, the complexity with limited vasculature [7, 241], fetal-transcriptome [242], relative lack of active synapses [232], and low-reproducibility [236] will be needed to be addressed in the future. Still, despite these limitations, 3D cerebral organoids have demonstrated in the last few years to be a powerful toolbox with the unique potential to explore novel therapeutic and genetic targets of AD.

## Conclusions

Taken together, iPSC-based models of AD have, thus far, provided numerous clues on molecular mechanisms that precede the development of AD pathology or confirmed those obtained from other model systems. While most of the studies focused on studying the AD-affected neurons, essential data was also acquired from investigations that focused on neural stem cells, glia, and cerebral organoids. Most importantly, the ability of these in vitro cultures to react to drug treatment provides hope that these models will be relevant on the way to finding a much-needed Alzheimer’s disease-modifying drug treatment. Finally, possible combination of iPSC-based models with various other approaches (i.e., in vitro models, in vivo models, medical imaging, biomedical markers) regarding their biological, genetic, and pathological similarities opens the door to further improve our understanding of Alzheimer´s disease.

## Data Availability

Not applicable.
